# Supplementary material for: Causal Relationship between Aging and Anorexia Nervosa: A White-Matter-Microstructure-Mediated Mendelian Randomization Analysis
Source: Biomedicines. 2024 Aug 16;12(8):1874. doi: 10.3390/biomedicines12081874 (PMC11351342; doi:10.3390/biomedicines12081874)
Supplement: Supplementary file 1 [file biomedicines-12-01874-s001.zip › Supplementary Figure/Figure S7/Single SNP plot of MR analysis for ubm-b-1985 to AN.pdf]

−10 0 10

MR effect size for

'IDP_dMRI_TBSS_OD_Medial_lemniscus_R || id:ubm_b_1985' on 'GCST[illegible]
